# A dental myth bites the dust – no observable relation between the incidence of dental abscess and the weather and lunar phase: an ecological study

**DOI:** 10.1186/s12903-015-0001-2

**Published:** 2015-02-11

**Authors:** Oliver Ristow, Steffen Koerdt, Ruben Stelzner, Matthias Stelzner, Christoph Johannes, Melanie Ristow, Bettina Hohlweg-Majert, Christoph Pautke

**Affiliations:** Department of Oral and Maxillofacial Surgery, University of Heidelberg, Im Neuenheimer Feld 400, D-69120 Heidelberg, Germany; Medizin & Aesthetik, Clinic for Oral and Maxillofacial and Plastic Surgery, Lenbachplatz 2a, D-80333 Munich, Germany; Department of Oral and Maxillofacial Surgery, Technical University Munich, Ismaninger Str. 22, D-81675 Munich, Germany; AllDent Zahnzentrum, Medizinisches Versorgungszentrum für Zahnheilkunde und Mund-, Kiefer-, Gesichtschiurgie, Bayerstr. 21, 80335 Munich, Germany; Private Statistical Consultant, Stuberstraße 16, D-80638 Munich, Germany; Department of Oral and Maxillofacial Surgery, Ludwig-Maximilians University Munich, Lindwurmstr. 2a, D-80336 Munich, Germany

**Keywords:** Weather, Meteorological medicine, Biosynoptic, Odontogenic abscess, Tooth-related infection, Moon, Lunar cycle

## Abstract

**Background:**

Anecdotal reports assert a relationship between weather and lunar activity and the odontogenic abscess (OA) incidence, but this relationship has not been validated. Therefore, the present study investigated the relationship between oral pain caused by OA and a variety of meteorological parameters and cyclic lunar activity.

**Methods:**

The records of all dental emergency patients treated at the AllDent Zahnzentrum Emergency Unit in Munich, Germany during 2012 were retrospectively reviewed. Patients with oral pain who were diagnosed with OA and treated surgically (n = 1211) were included in the analysis. The OA incidence was correlated to daily meteorological data, biosynoptic weather analysis, and cyclic lunar activity.

**Results:**

There was no seasonal variation in the OA incidence. None of the meteorological parameters, lunar phase, or biosynoptic weather class were significantly correlated with the OA incidence, except the mean barometric pressure, which was weakly correlated (rho = -0.204). The OA incidence showed a decreasing trend as barometric pressure increased (p < 0.001). On multiple linear regression, the barometric pressure accounted for approximately 4% of the OA incidence.

**Conclusion:**

There is no evidence supporting a correlation between the incidence of odontogenic abscess and the weather and lunar activities.

## Background

Tooth abscess comprises a substantial portion of dental and oral-maxillofacial emergencies. Although dental emergencies are unpredictable, several factors, including weather and lunar activity, are thought to affect the occurrence of odontogenic abscesses (OA). There is a widespread myth that odontogenic abscess changes with variations in weather activity. High temperature and barometric pressure changes are often implicated as causative factors, and many physicians correlate changing weather conditions with a high incidence of abscess, particularly in German-speaking countries [1-3]. A few investigative studies were conducted during the early 1970s, but overall, evidence-based studies are surprisingly sparse, and most studies are inadequate, reporting gross numbers (inconsistent abscess incidences), evaluating small sample populations, or examining a limited set of weather parameters [1,2,4]. A recent study of 1090 patients found a correlation between oral pain and barometric pressure changes, but the correlation to OA was not examined [3].

Controversy surrounds investigations of the potential correlations between lunar phases and clinical pathologies such as wound healing or pain [5]. More than 10% of the German population believes that there is a relationship between lunar activity and health [6], and many individuals exhibit a moon-dependent lifestyle with work activity decreased on specific days [7]. Studies have been conducted examining the relationship between lunar activity and myocardial infarction [8], postoperative morbidity [9], emergency admissions [5], and oral pain [3], but there are no known studies investigating the correlation between lunar activity and OA.

Therefore, the present study investigated the relationship between the OA incidence and daily weather patterns (meteorological parameters, biosynoptic weather analysis) and lunar activity.

## Methods

### Patients

We retrospectively reviewed the medical records of all patients examined for oral or dental pain at the AllDent Zahnzentrum Emergency Unit in Munich, Germany from January to December 2012. The sample population comprised patients who were diagnosed with an odontogenic abscess (OA) requiring surgical repair (n = 1211) [4]. Patients diagnosed with other conditions, including infection, iatrogenic infection, malignancy, fracture, or lesions arising from other locations, were excluded from the study. Patients who previously received antibiotic therapy or were previously diagnosed with infection were also excluded. This study followed the tenants of the Declaration of Helsinki on medical protocol and ethics. Due to the retrospective nature of this study, the requirement for ethical approval was waived in writing by the institutional review board of the University of Munich.

### Meteorological parameters

Weather data were obtained from the German Weather Service (Munich Airport station; German Federal Ministry of Transport, Building, and Urban Development). Data were acquired 24 times per day (once every hour) and were calculated to generate the daily mean. Temperature (°C) was measured using an electronic thermometer positioned 2 m above ground. The barometric pressure at station altitude was measured using a vibrant thread barometer (hPa). The relative humidity (%), total precipitation (mm), and total sunshine duration (h) were also recorded.

### Weather class

The weather classes were based on those described by Bucher et al. [10,11], which uses systemic biosynoptic weather analysis. Weather classification is primarily based on vorticity, and is physically and meteorologically substantiated by objective criteria. To refine this classification system, Bucher et al. [10,11] also included changes in the temperature-humidity milieu between the effective date and the preceding 7 days to generate five primary weather classes: class 1, anticyclonic; class 2, warm air advection; class 3, cyclonic maximum; class 4, cold air advection; and class 5, indifferent. All weather class data were calculated and provided by the Department of Meteorological Medicine, German Weather Service, Freiburg, Germany using the definitions described by Bucher et al. [10,11].

### Lunar phase

The lunar cycle was derived from an ephemeris and divided into four equal intervals: first quarter, full moon, last quarter, and new moon.

### Statistical analysis

The relationship between the OA incidence and the weather and lunar activities was examined using Spearman’s correlation coefficient. To adjust for potential delayed effects of barometric pressure on the OA incidence, the 3-day mean barometric pressure was calculated from the mean on the investigation day and the two previous days. The relationship between the OA incidence as a dependent variable and the weather and lunar activities as independent variables were investigated using multiple linear regression. All statistical analyses were performed using SPSS 21.0. The global alpha was set at 5%, and the local significance level was set at 0.006 after performing the Bonferroni adjustment for multiple testing.

## Results

A total of 1211 patients (539 [44.5%] women; 672 [55.5%] men; mean age ± standard deviation 43 ± 18.5 years; age range 18–75 years) were diagnosed with OA over the study period. The relative OA incidence (number of OA patients/number of emergency patients) was 3.5% in 2012 (range 2.8–4.5% per month). The mean daily OA incidence was 3.0% in 2012 (range 2–5 per month). There was no statistically significant variation in the mean month, weekly, or daily incidence of OA (p > 0.05).

**Figure 1:**
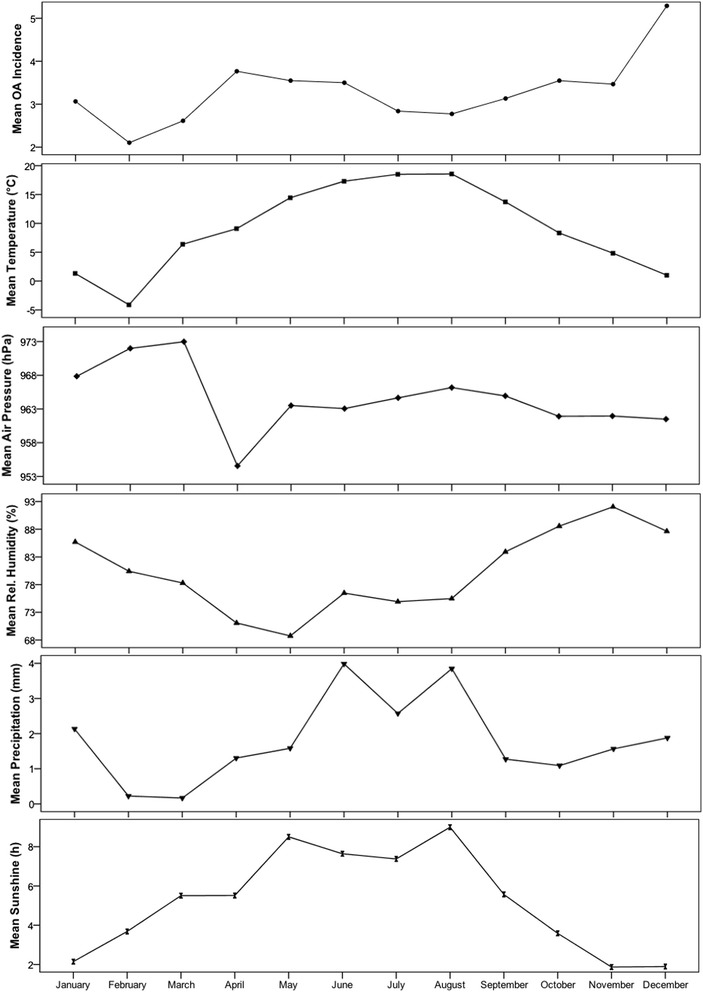
**Relationship between the OA incidence and daily weather conditions during January to December 2012.** The mean incidence of odontogenic abscess (OA) was examined according to the mean daily temperature (°C), barometric pressure (hPa; daily mean and 3-day mean), and sunshine duration (h), and the biosynoptic weather class (class 1, anticyclonic; class 2, warm air advection; class 3, cyclonic maximum; class 4, cold air advection; and class 5, indifferent).

**Figure 2:**
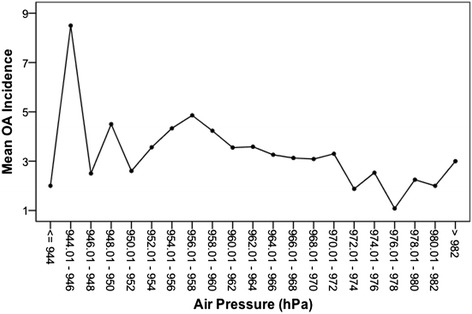
**Relationship between the OA incidence and mean daily barometric pressure.** The mean odontogenic abscess (OA) incidence per equidistant air pressure (hPa) interval is indicated. The interval lengths were chosen arbitrarily to simplify delineation. A statistically significant (p = 0.001) but weak correlation was observed, but the small correlation coefficient (rho = -0.204) does not support a sound relationship.

As shown in Table 1, there was no statistically significant correlation between the OA incidence and the mean temperature, relative humidity, total precipitation, or total sunshine duration (p > 0.05). The variation in OA incidence occurred independently of the weather activity (Figure 1). However, there was a statistically significant weak correlation (p = 0.0001, rho = -0.204) between the OA incidence and barometric pressure (Figure 2). The OA incidence decreased as the barometric pressure increased, and the highest OA incidence occurred at a barometric pressure of 942.0 hPa, while the lowest occurred at 981.0 hPa. When the correlation was examined between the OA incidence and the 3-day mean barometric pressure, we observed a similar trend. While there was a statistically significant weak correlation between the barometric pressure and OA incidence (p = 0.0001), a sound dependent relationship was not observed, as indicated by the small coefficient (rho = -0.204). When the relationship was subjected to multiple linear regression, we observed that the barometric pressure accounted for approximately 4% of the OA incidence.

**Table 1:** **Relationship between the incidence of odontogenic abscess and daily weather conditions or lunar activity by Spearman’s rho correlation coefficient**

**Variable**	**Correlation coefficient**	**p-value**
Barometric pressure	−0.204	0.0001*
3-day mean barometric pressure	−0.203	0.0001*
Sunshine duration	−0.076	0.149
Biosynoptic weather class	−0.055	0.291
Lunar phase	−0.048	0.360
Precipitation	0.034	0.514
Relative humidity	0.034	0.523
Temperature	−0.020	0.700

*Significance at p < 0.006 (after Bonferroni adjustment for multiple testing) using Spearman’s rho correlation coefficient. Notably, the mean daily barometric and 3-day mean barometric pressure were correlated because the 3-day parameter includes daily barometric pressure data.

**Figure 3:**
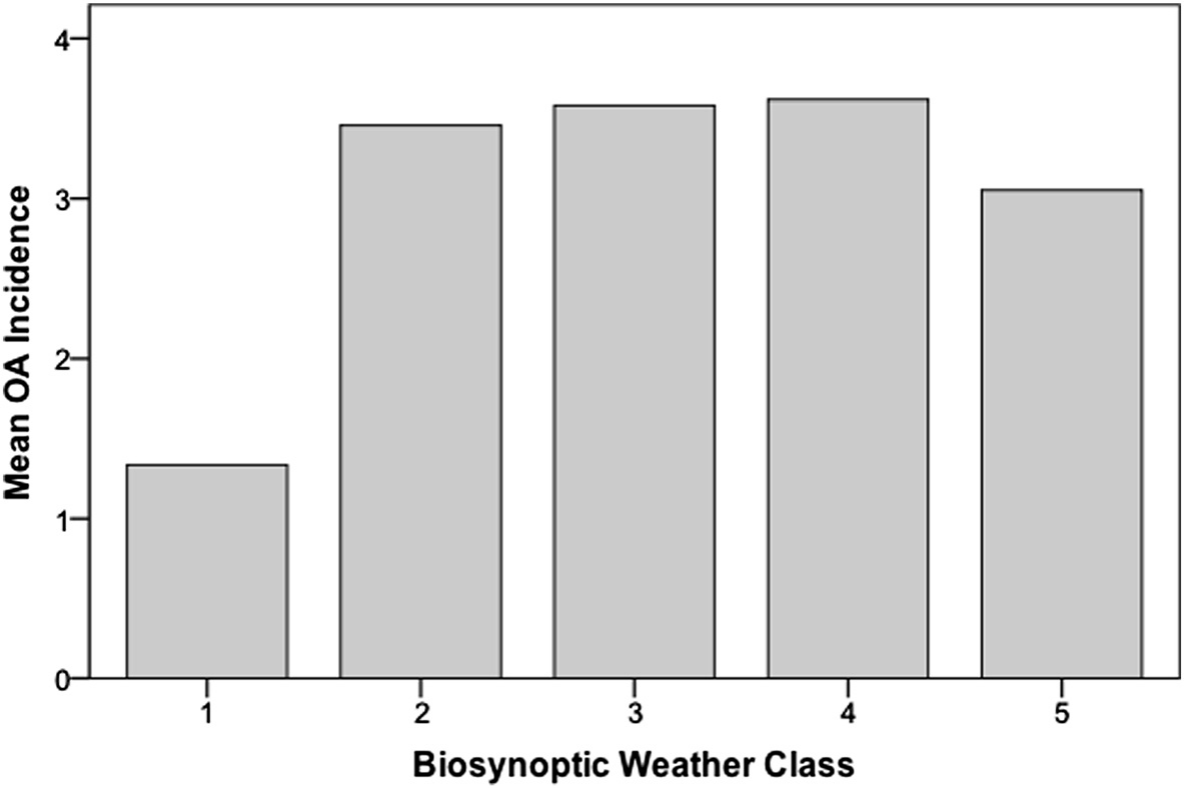
**Relationship between the OA incidence and biosynoptic weather class.** Weather was classified on a scale from 1 to 5 (class 1, anticyclonic; class 2, warm air advection; class 3, cyclonic maximum; class 4, cold air advection; and class 5, indifferent). There was no statistically significant correlation observed (p > 0.05).

The OA incidence did not show any significant correlation to the biosynoptic weather class (p > 0.05, Table 1). The mean OA incidence remained steady at classes 2 to 5. Although the mean OA incidence was smaller for class 1 (Figure 3), the differences between the classes were not statistically significant (p > 0.05). Notably, class 1 weather only appeared on four days during 2012. There was no significant correlation between the lunar phase and OA incidence (p > 0.05, Table 1).

When all the weather and lunar phase data were included in the regression model, the explained variance improved only slightly from R^2^ = 0.039 to R^2^ = 0.058. The temperature, relative humidity, precipitation, sunshine, lunar phase, and biosynoptic weather class had no influence on the OA incidence (Table 2).

**Table 2:** **Relationship between the incidence of odontogenic abscess and daily weather conditions or lunar activity by multiple linear regression analysis**

**Variable**	**Coefficient**	p-value*
Barometric pressure	−0.179	0.002
Sunshine duration	−0.137	0.083
Relative humidity	−0.086	0.252
Lunar phase	−0.032	0.538
Biosynoptic weather class	−0.006	0.919
Precipitation	−0.003	0.958
Temperature	−0.002	0.974

*Significance at p < 0.006 (after Bonferroni adjustment for multiple testing).

## Discussion

A relationship has long been suspected between environmental conditions and human disease [12-15]. There is a strong prevailing belief among dentists and oral surgeons that certain weather conditions influence the incidence of oral pain caused by tooth and oral abscesses. Several studies have examined the effect of barometric pressure on oral pain (barodontalgia), particularly during extreme conditions such as diving, flying, or mountaineering [16-18]. Though pain is primarily a subjective phenomenon, a psychosomatic component cannot be excluded in persons.

Kloss-Brandstätter et al. recently postulated that the correlation between atmospheric pressure changes and oral pain was a daily phenomenon where patients were unaware of daily barometric changes [3]. However, the study evaluated pain as the primary endpoint and did not distinguish between the various etiologies (such as caries, periodontitis, and pericoronitis). It was also limited to a 3-month period. It remains unclear whether the environmental influence on human health is purely subjective or if weather conditions actually trigger inflammation or change disease activity. Therefore, in the present study, we solely evaluated the incidence of OA as an indicator of oral pain.

We did not observe any relationship between the OA incidence and the mean daily temperature, relative humidity, total precipitation, total sunshine duration, or biosynoptic weather class. Similarly, we did not detect any correlation between the lunar phase and OA occurrence. However, we did observe a decreasing trend in the OA incidence during periods of increasing barometric pressure. These results mirror those in earlier studies, which report that the OA incidence increases significantly during periods of low barometric pressure [1,2], though no causative relationship has been reported. However, correlation does not necessarily indicate causation; two variables may be related to each other, but this does not confirm that one variable causes the other. Statistical correlation tends to strengthen in a large sample population; thus, the weak correlation coefficient in the present study (rho = -0.204) does not support a dependent relationship between barometric pressure and OA.

Few studies have examined the relationship between weather conditions and odontogenic abscesses, and the results are contradictory [1,2,4]. There are no known studies investigating the correlation between weather condition and OA incidence over as long a period (1 year) as in the present study.

Meningeaud et al. investigated the relationship between the monthly incidence of odontogenic cellulitis and the mean monthly temperature and mean monthly barometric pressure over a 12-month period. Their results suggested that the occurrence of odontogenic cellulitis was not influenced by temperature and atmospheric pressure [4], which is consistent with the present findings. However, due to their small sample population, the mean incidence of odontogenic cellulitis was averaged on a monthly basis. Because weather constantly changes, the influence of daily weather variations on the OA incidence may have been overlooked. In addition, the study was limited to only two meteorological parameters.

Schuld et al. sought stronger evidence by using multiple clinical settings with large patient populations and examining cyclic phenomena (e.g. weather, lunar phase, and season) [19]. However, geographic diversity generates meteorological discrepancies, resulting in a complex and difficult analysis. We also sought strong evidence, but instead used data from the AllDent Zahnzentrum Emergency Unit in Munich, Germany, which is the only facility providing 24/7 emergency dental service in Munich. This ensured a steady daily flow of cases irrespective of the month or day, making calendar adjustments unnecessary. Additional studies are needed to examine meteorological regions other than Munich, which have different thermal and weather conditions.

The present study is limited in its ability to accurately measure the incidence of OA in the local population. The incidence of OA was based on the frequency of dental examination at a single center rather than the population-wide incidence of OA and acute oral pain. In addition, environmental conditions may have influenced patient behavior and the decision to seek medical intervention. The relationship between weather condition, patient behavior, and the measured OA incidence is difficult to quantify; however, every effort was made to generate an accurate incidence in the present study.

## Conclusion

This study does not evaluate how environmental factors influence subjective pain, inflammation, or disease activity, but it does debunk a widespread myth among dentists and oral surgeons: namely, there is no evidence of any correlation between weather conditions or lunar activity and the incidence of oral pain caused by odontogenic abscess.
